# Back to the Wild: On a Quest for Donors Toward Salinity Tolerant Rice

**DOI:** 10.3389/fpls.2020.00323

**Published:** 2020-03-20

**Authors:** Celymar A. Solis, Miing T. Yong, Ricky Vinarao, Kshirod Jena, Paul Holford, Lana Shabala, Meixue Zhou, Sergey Shabala, Zhong-Hua Chen

**Affiliations:** ^1^School of Science, Western Sydney University, Penrith, NSW, Australia; ^2^Tasmanian Institute of Agriculture, University of Tasmania, Hobart, TAS, Australia; ^3^International Rice Research Institute, Metro Manila, Philippines; ^4^International Research Centre for Environmental Membrane Biology, Foshan University, Foshan, China; ^5^Hawkesbury Institute for the Environment, Western Sydney University, Penrith, NSW, Australia

**Keywords:** wild rice, salinity stress, molecular breeding, rice domestication, *O. coarctata*, *O. rufipogon*

## Abstract

Salinity stress affects global food producing areas by limiting both crop growth and yield. Attempts to develop salinity-tolerant rice varieties have had limited success due to the complexity of the salinity tolerance trait, high variation in the stress response and a lack of available donors for candidate genes for cultivated rice. As a result, finding suitable donors of genes and traits for salinity tolerance has become a major bottleneck in breeding for salinity tolerant crops. Twenty-two wild *Oryza* relatives have been recognized as important genetic resources for quantitatively inherited traits such as resistance and/or tolerance to abiotic and biotic stresses. In this review, we discuss the challenges and opportunities of such an approach by critically analyzing evolutionary, ecological, genetic, and physiological aspects of *Oryza* species. We argue that the strategy of rice breeding for better Na^+^ exclusion employed for the last few decades has reached a plateau and cannot deliver any further improvement in salinity tolerance in this species. This calls for a paradigm shift in rice breeding and more efforts toward targeting mechanisms of the tissue tolerance and a better utilization of the potential of wild rice where such traits are already present. We summarize the differences in salinity stress adaptation amongst cultivated and wild *Oryza* relatives and identify several key traits that should be targeted in future breeding programs. This includes: (1) efficient sequestration of Na^+^ in mesophyll cell vacuoles, with a strong emphasis on control of tonoplast leak channels; (2) more efficient control of xylem ion loading; (3) efficient cytosolic K^+^ retention in both root and leaf mesophyll cells; and (4) incorporating Na^+^ sequestration in trichrome. We conclude that while amongst all wild relatives, *O. rufipogon* is arguably a best source of germplasm at the moment, genes and traits from the wild relatives, *O. coarctata*, *O. latifolia*, and *O. alta*, should be targeted in future genetic programs to develop salt tolerant cultivated rice.

## Introduction

Rice (*Oryza sativa* L.) is one of the most important staple crops in many countries. It is the main food source of over half of the world population accounting for about 50–80% of their daily calorie intake (International Rice Genome Sequencing Project, 2005; Food and Agriculture Organization, 2019). With an increasing world population, there is a need to increase rice production by 87% by 2050 (Kromdijk and Long, 2016). This is a challenging task, as the amount of arable land available to meet this demand for the increase in rice production is simply insufficient using current practices. Moreover, a large proportion of the global food production will have to deal with erratic environmental conditions and abiotic stresses due to climate change (Food and Agriculture Organization, 2019).

It is estimated that approximately 950 million hectares of arable land globally, including 250 million hectares of irrigated land, is affected by salinity (Yamaguchi and Blumwald, 2005; Shahbaz and Ashraf, 2013). Among the cereal crops, rice is the most sensitive to salinity, and high salt concentrations in soils are considered as one of the major abiotic stresses affecting rice production (Eynard et al., 2005). Rice is grown in over 100 countries in an area of more than 150 million hectares of land; however, a large proportion of these agricultural areas are salinized to some degree or are at risk of being salinized. This involves millions of hectares in South Asia and Southeast Asia that are climatically suited for rice production but are left uncultivated or have low yields due to saline soils (Smajgl et al., 2015). However, the demand for rice is rising due to increasing populations in countries where rice is the staple food and the growing global popularity of rice cuisines. Given the economic significance of rice, understanding the mechanisms of adaptation to salinity stress and, subsequently, developing salinity-tolerant genotypes can be a means of increasing rice production. Major efforts have been made to increase the salt tolerance of rice through conventional and molecular breeding, but the extent of increase in salt tolerance has been limited, especially under field conditions (Hoang et al., 2016).

Wild *Oryza* species are a prime reservoir of genetic diversity for the improvement of one the world’s most important food crops. The genus is composed of two domesticated species, *Oryza sativa* and *Oryza glaberrima*, and 22 wild species that represent between 15 and 25 million years of evolutionary diversification (Vaughan, 1994). Wild rice relatives have been identified as potential sources of important agronomic traits. Modern breeding approaches to stress tolerance have been focused on the identification of the sources of genetic diversity in the primary gene pool and then introgressing favorable alleles into elite cultivars to combat the stresses (Gur and Zamir, 2004; Sharma et al., 2013). Introgression of favorable alleles into elite rice varieties has been successful for development of lines that are resistant to pests and diseases (Jena and Kim, 2010; Sarao et al., 2016) and higher yields (Swamy and Sarla, 2008; Bai et al., 2012), but to a less extent for tolerance to drought, cold and salinity (Koseki et al., 2010; Ndjiondjop et al., 2010; Yang et al., 2012).

In this review, we discuss the genus *Oryza* and its genetic complexity and the limitations in the potential use of these wild rice species, with a focus on breeding of salinity-tolerant rice. We elucidate the variability in response to salinity amongst *Oryza* species and propose some key genes from wild rice as targets for further research toward the identification of salt tolerance mechanisms in wild rice species for the improvement of cultivated rice.

## The Complex *Oryza* Genus and the Evolution and Domestication of Modern Cultivated Rice

The genus, *Oryza*, has a rich genetic diversity that comprises different landraces, modern and obsolete varieties, genetic stocks, and wild *Oryza* species (Zhang and Gao, 2017). Modern cultivated rice has evolved from its wild progenitors through a series of introgressive events, natural selection, domestication, and conventional and molecular breeding. Given these processes, just two of the 24 extant *Oryza* species have become agronomically productive: “Asian rice,” *Oryza sativa*, that is high yielding and cultivated worldwide and “African rice,” *Oryza glaberrima*, that is low yielding and grown in limited areas in West Africa (Vaughan, 1994; Linares, 2002; Vaughan et al., 2003). The domestication of these two cultivated rice species shows parallel evolution in which different human civilizations selected different wild species to target the same traits (Purugganan, 2014). These traits resulted in major alterations in rice plant structure and reproductive physiology. Rice breeders have selected for the characteristics of rice grains that make them more appealing as a food source, including grain size, shape, color, and fragrance (Kovach et al., 2007). Despite being widely studied, the domestication of these two cultivated species remains controversial (Wambugu et al., 2018). Modern genetic and genomic studies of cultivated and wild rice will provide useful information and vital insights into the evolution and domestication of rice (Kovach et al., 2007).

**FIGURE 1:**
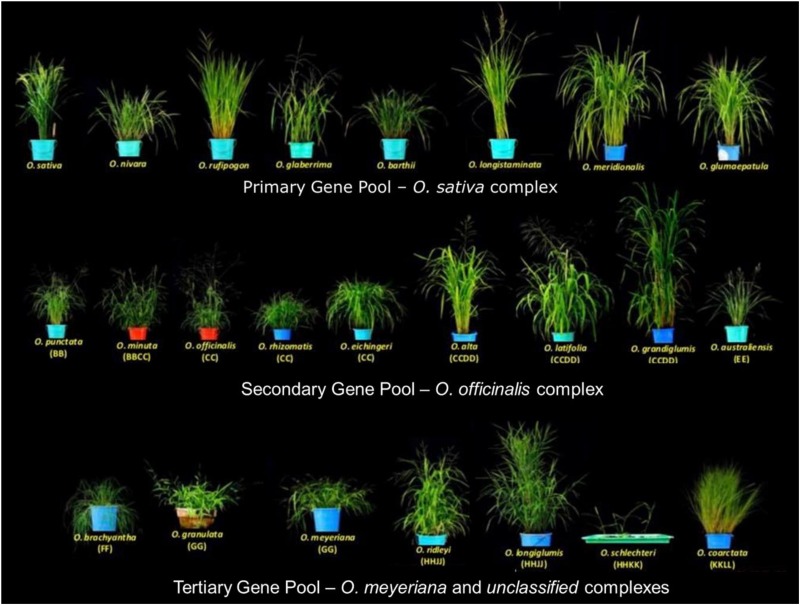
Diversity of wild rice species. The 24 wild *Oryza* species are divided into a primary gene pool (*Oryza sativa* complex, AA genome), a secondary gene pool (*Oryza officinalis* complex, BB to EE genomes), and a tertiary gene pool (*O. meyeriana* complex and unclassified species, FF to KKLL genomes). Images are taken by the authors at the International Rice Research Institute (IRRI).

**FIGURE 2:**
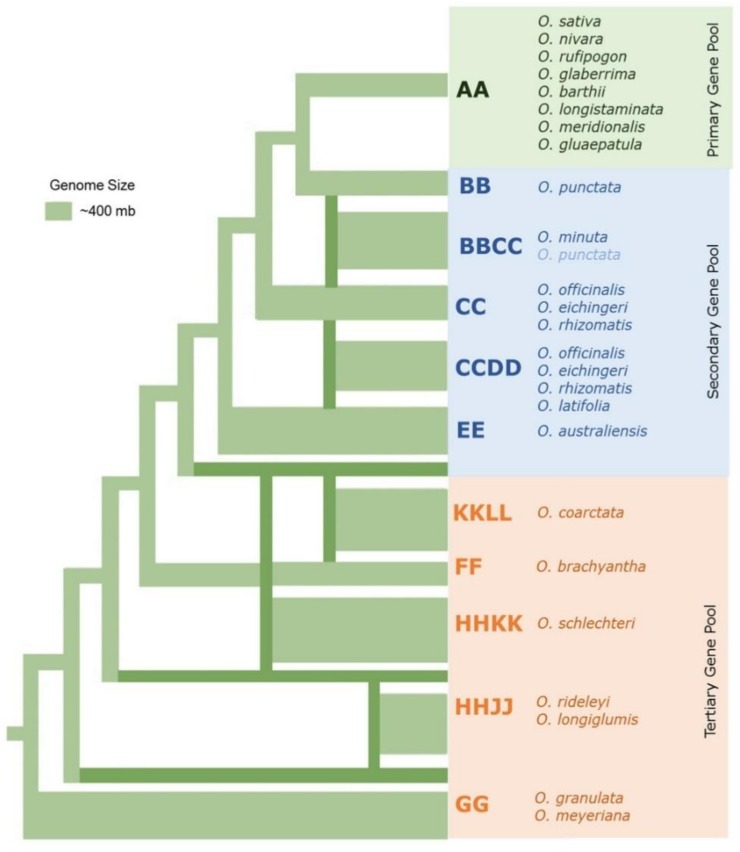
The phylogenetic relationships among the major wild rice species. Neighbor joining tree in which the thickness of the lines connecting to each lineage represents approximate genome size modified from Ge et al. (1999); Lu et al. (2009), and Ammiraju et al. (2010).

The 22 wild species have native distributions in tropical and subtropical regions of Africa, South-East Asia, Australasia, and Central and South America (Supplementary Table S1). These wild rice relatives are weedy grasses (Figure 1) that have inferior morpho-physiological characteristics, poor grain attributes, and low seed set compared to cultivated rice. These wild rice species also differ substantially in their growth habitat and morphological characteristics such as plant height, leaf shape and size, tiller numbers, flowering, panicle size, and branching (Vaughan et al., 2003). Fifteen of the 22 wild *Oryza* species have been assigned to six diploid genome types (AA, BB, CC, EE, FF, and GG) and the remaining seven species to three tetraploid species types (BBCC, CCDD, and HHJJ) (Figure 2). These types are determined based on cytogenetic analysis (Morinaga, 1964), genomic DNA hybridization (Aggarwal et al., 1997), rDNA spacers (Cordesse et al., 1992), transposons (Iwamoto et al., 1999; Kanazawa et al., 2000), and DNA sequence comparisons of two *Adh* genes (Ge et al., 1999). Although the genome of *O. sativa* (AA) has been fully determined, many aspects of the evolution of the *Oryza* genus and their phylogenetic relationships (Stein et al., 2018) remain to be clarified.

The genus *Oryza* can be grouped into four species “complexes”: (1) the primary gene pool, the *O. sativa* complex; (2) the *O. officinalis* complex; (3) the *O. ridleyi* complex; and (4) the *O. meyeriana* complex (Supplementary Table S1). The *O. sativa* complex comprises eight diploid species with AA genomes that encompasses two cultivated (*O. sativa* and *O. glaberrima*) and six wild rice species (*O. nivara*, *O. rufipogon*, *O. breviligulata*, *O. longistaminata*, *O. meridionalis*, and *O. glumaepatula*). These species are commonly used by breeders due to their ease of crossing and gene transfer to cultivated rice cultivars (Brar and Khush, 2018). The most widely used ancestral wild rice, *O. rufipogon*, is found in regions of South Asia, Southeast Asia and Northern Australia, and has both annual and perennial types. Perennial *O. rufipogon* subtypes are found in deep swamps, while the annual subtypes are found in shallower, temporary swamps which are parched in the dry season (Morishima et al., 1984). *O. rufipogon* has been used for many decades for the breeding of disease- and insect-resistant rice (Song et al., 2005; Stein et al., 2018; Wing et al., 2018). *O. nivara* is an annual grass within the *O. sativa* complex that is distributed in India, Nepal, Cambodia, Laos, and Thailand. As with *O. rufipogon*, it has been used to breed high-yielding, virus-resistant rice cultivars (Varshney et al., 2009; Brar and Singh, 2011; Swamy et al., 2014). A few introgression lines have been developed using yield-associated QTLs from *O. nivara* (Ma et al., 2016). *Oryza longistaminata*, a perennial plant that is widely distributed in Africa, is closely related to its annual relative, *O. barthii* (Akimoto et al., 1998). These two species are closely related to *O. glaberrima*, which mainly distributed in Western Africa in upland, rain-fed and deep-water fields (Linares, 2002; Wang et al., 2014). *Oryza glaberrima* can be easily distinguished from *O. sativa* due to its short, rounded ligule and no secondary panicle branching (Vaughan et al., 2003). *Oryza glaberrima* is tolerant to different biotic and abiotic stresses, but its yield is not high (Futakuchi et al., 2012).

The O. officinalis complex comprises six diploid species (*O. punctata*, *O. minuta*, *O. officinalis*, *O. eichingeri*, *O. rhizomatis*, *O. australiensis*) and six allotetraploid species (*O. punctata*, *O. minuta*, *O. officinalis*, *O. latifolia*, *O. alta*, and *O. grandiglumis*) (Brar and Khush, 2018). This complex is distributed in Asia, Africa, and Latin America. In Asia, the most common species are *O. minuta* and *O. officinalis* (Vaughan, 1989); *O. punctata* (BB, annual, BBCC perennial) and *O. eichngeri* (CC) are mainly found in Africa (Vaughan et al., 2003). The three allotetraploids, *O. latifolia*, *O. alta*, and *O. grandiglumis* (CCDD), are found in the Latin America. *Oryza latifolia* is widely distributed in Central and South America and the Caribbean islands while *O. alta* and *O. grandiglumis* grows in the Amazon basin of South America (Bao and Ge, 2004). *Oryza australiensis* (EE) is commonly found in northern parts of Australia and survives as both rhizomes and seeds. This species has the largest genome in the genus, *Oryza*, due to a retrotransposon (Piegu et al., 2006).

The *O. ridleyi* complex comprises two tetraploid (HHJJ genomes) species, *O. ridleyi* and *O. longiglumis*, that are commonly found in Asia and have highly similar morphological and ecological characteristics. Rice breeders have used these species to breed cultivars resistant to bacterial blight and blast (Vaughan et al., 2003). The *O. meyeriana* complex is composed of two species, *O. meyeriana* and *O. granulata*, both with diploid GG genomes. Compared to species in the other complexes, these plants are small with unbranched panicles and small spikelets. They thrive in shady or partial shady forest understory and cannot survive flooding (Vaughan et al., 2003). Two of the 24 species are yet to be classified among the complexes: *O. schleteri* (HHKK), which is distributed in regions of Indonesia and Papua New Guinea; and *O. coarctata* (KKLL), which is commonly found in coastal areas of South Asia (Vaughan et al., 2003).

## Search for Donors for Salt Tolerant Rice

There are numerous rice varieties widespread across the globe, with over 127,000 reported accessions of cultivated rice and wild relatives (Supplementary Table S1). Among the known accessions, there are thousands of hybrid rice cultivars that have characterized quantitative traits such as grain yield, flowering time and plant architecture. There are also classic elite cultivars, such as IR64, Teqing and IR68552-55-3-2, that are used as recurrent parental lines (Huang et al., 2016). However, only a small proportion of these natural accessions have been assessed for traits under saline conditions using different screening approaches and experimental designs (Platten et al., 2013; Kumar et al., 2015). It has been found that the salt tolerance of cultivars of *O. glaberrima* and *O. sativa* is related to low Na^+^ and high K^+^ concentrations in roots and shoots (Reddy et al., 2014; Rahman et al., 2016; Sakina et al., 2016), and some of the most salt-tolerant cultivars are Pokkali, Nona Bokra, and FL478 (Zeng and Shannon, 2000; Platten et al., 2013). These salt-tolerant cultivars have been extensively used in breeding salinity-tolerant rice (Thomson et al., 2010; Platten et al., 2013; Reddy et al., 2014; Waziri et al., 2016). However, the degree of salt tolerance has often been low, and the developed plants possess tolerance like the donors but with poor agronomical traits. Most of these poor agronomical traits are linked with salinity tolerance, making it difficult to untangle them and produce tolerant lines while avoiding linkage drag of these poor agronomic traits from the donors (Gregorio et al., 2002). Also, the developed lines often survived only under glasshouse conditions and failed to show salinity tolerance in the field due to combined stresses (Reddy et al., 2014). Thus, it appears that the extent of genetic variability in salinity tolerance in cultivated rice is not large enough to enable any significant improvement in this trait and create varieties capable of handling the substantial amounts of salt that can be present in the rhizosphere.

Compared to cultivated rice, the wild rice species have greater genetic diversity. It is estimated that 10–20% of wild rice species diversity is represented in cultivated rice (Zhu et al., 2007; Stein et al., 2018). The large genetic diversity suggests that novel salinity tolerance mechanisms can be explored for the potential utilization of genes and traits contained in this diversity. Thus, an attractive breeding strategy could be to combine salinity-adaptive traits from wild rice through gene pyramiding in popular varieties and elite breeding lines (Gregorio et al., 2002).

## Mechanisms of Adaptation to Salinity Stress in Rice

### Physiological Mechanisms of Rice Salt Tolerance

Rice is the most salinity-sensitive species among the cereal crops, where 3 dSm^–1^ of salinity is sufficient to cause significant yield reductions in most cultivated varieties (Eynard et al., 2005). The salt tolerance of rice varies at different growth stages. The crop is relatively tolerant during seed germination (Khan et al., 1997) and the early vegetative stage (shoot and root emerge until the first tiller grows) (Zeng and Shannon, 2000), but it is susceptible at the seedling stage (Lutts et al., 1996) and highly susceptible at the reproductive stage (Khatun and Flowers, 1995). The major morphological symptoms in salinity-sensitive rice varieties include stunted roots and shoots, leaf tip burning, low tiller numbers, reduced numbers of florets per panicle, low pollen viability, spikelet sterility, reduced panicle numbers, low grain weight, and low harvest index and yield (Zeng and Shannon, 2000; Singh et al., 2008).

**FIGURE 3:**
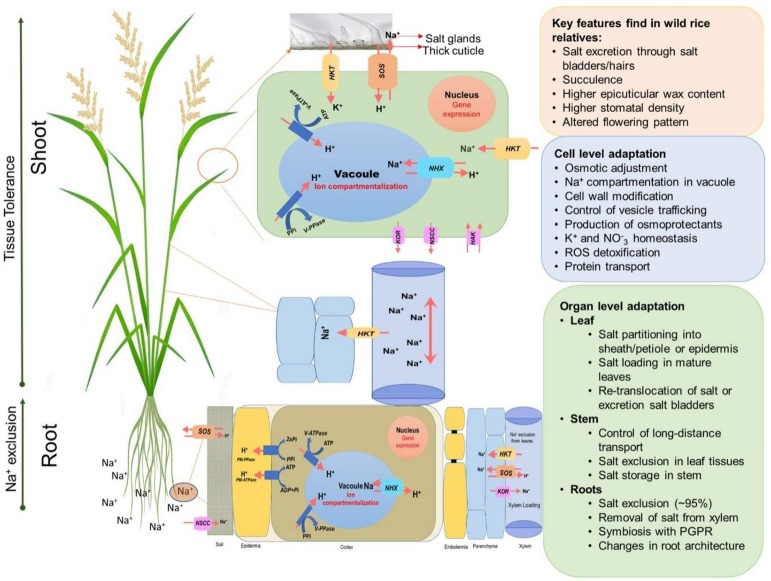
An overview of adaptive mechanisms of rice species under salinity stress. Diagram shows the main salt tolerance mechanisms such as Na^+^ exclusion in the roots, enhanced xylem Na^+^ unloading, Na^+^ compartmentalization in vacuoles, Na^+^ exclusion in leaf blades/salt glands.

Plants generally adapt to salinity stress by mechanisms related to ion exclusion and osmotic tolerance (Figure 3) (Munns and Tester, 2008; Reddy et al., 2017; Munns et al., 2020). Ion exclusion is traditionally considered to be related to minimizing the initial entry of salt into roots and/or its efficient retention in the roots thus preventing excess accumulation of Na^+^ and Cl^–^ ions in the leaves (Roy et al., 2014). As a result of selective breeding, salt-tolerant rice varieties accumulate less Na^+^ in leaves and shoots than those of salt-sensitive cultivars (Golldack et al., 2003; Lee et al., 2003; Lin et al., 2004; Ren et al., 2005; Cotsaftis et al., 2012). Ion exclusion also includes the retrieval of Na^+^ into xylem parenchymal cells and the efflux of ions back into the soil. Osmotic tolerance is related to a plant’s ability to increase production of organic osmolytes and/or the use of inorganic salts for osmotic adjustment. This osmotic adjustment is critical to maintain leaf expansion and operate stomatal movements (Roy et al., 2014). A plant’s reliance on inorganic ions for osmotic adjustment implies efficient vacuolar Na^+^ compartmentation (Shabala et al., 2019) and represents an important component of the tissue tolerance mechanism, e.g., an ability to maintain optimal functioning in the presence of high Na^+^ and Cl^–^ in plant tissues. This requires compartmentalization of Na^+^ and Cl^–^ at the cellular and intracellular levels to avoid toxic concentrations within the cytoplasm (Chen et al., 2007b; Shabala et al., 2019), especially in leaf mesophyll cells. As a result of this sequestration, plants are capable of maintaining a high tissue K^+^/Na^+^ ratio that is considered a key determinant of salinity stress tolerance (Maathuis and Amtmann, 1999; Anschütz et al., 2014; Shabala and Pottosin, 2014). In addition, plants use compatible solutes and produce enzymes catalyzing the detoxification of reactive oxygen species (ROS) (Bartels and Sunkar, 2005; Chen et al., 2007a) to deal with the oxidative component of the salt stress.

### Molecular Mechanisms for Rice Salt Tolerance

Salinity tolerance is a complex, quantitative trait that is controlled by multiple factors, and many genes have been reported to enhance salt tolerance in rice. These genes fall in three categories: (1) genes for salt uptake, transport and signaling; (2) genes for osmoprotection; and (3) genes for maintaining energy balance for normal growth under salinity stress (Munns et al., 2020). Figure 3 shows some of the key genes involved in salt tolerance that are likely to be relevant for controlling K^+^ or Na^+^ uptake by roots and transport within the plant. Na^+^ uptake in the roots from soil is mediated primarily by non-selective cation channels (NSCCs) that have similar permeability to Na^+^ and K^+^ (Demidchik et al., 2002) and through K^+^ transporters (HKTs) that mediate Na^+^ and K^+^ transport (Tester and Davenport, 2003; Plett and Møller, 2010; Hanin et al., 2016). The Na^+^ absorbed by the root moves to the xylem via other transporters and channels and is delivered to the shoots (Munns and Tester, 2008). Regulating transport of Na^+^ is crucial in salinity stress adaptation in rice. *OsHKT1;5* (*SKC1*) is responsible for Na^+^ exclusion in the vasculature for protecting leaf blades and reproductive tissues from salt toxicity in rice (Kobayashi et al., 2017). *SKC1* is preferentially expressed in parenchyma cells surrounding xylem vessels for efficient recirculation of Na^+^ by unloading Na^+^ into the xylem thereby decreasing Na^+^ accumulation in shoots, contributing to increased salinity tolerance (Ren et al., 2005). Similarly, the rice K^+^ transporter protein, *OsHKT1;4*, a non-selective transporter, controls the uptake of Na^+^ during the reproductive growth stage (Suzuki et al., 2016), and *OsHKT1;1* is associated with the regulation Na^+^ accumulation in shoots where it is mainly expressed in the phloem of leaf blades and is up-regulated in response to salinity stress (Garciadeblás et al., 2003).

The efficient exclusion of Na^+^ excess from the cytoplasm and vacuolar Na^+^ accumulation is the most important step toward the maintenance of ion homeostasis inside the cell. The Na^+^/H^+^ antiporters, SOS1 and NHX1, are the key genes involved in active Na^+^ extrusion to the apoplast and Na^+^ compartmentation in the vacuole, respectively (Figure 3). Both antiporters are driven by a proton-motive pump force generated by H^+^-ATPase (Blumwald, 2000). Overexpression of SOS1 of multiple plant species leads to salt tolerance in plants (Shi et al., 2000; Pardo et al., 2006; Oh et al., 2009; Olías et al., 2009). An *OsSOS1* loss of function mutant displayed exceptional salt sensitivity that was correlated with excessive Na^+^ uptake and impaired Na^+^ loading into the xylem (El Mahi et al., 2019). NHXs sequester Na^+^ into vacuoles and are used for osmotic adjustment in salt tolerant plants (Apse et al., 1999; Nieves-Cordones et al., 2016). NHXs mediate Na^+^ influx in exchange for H^+^ efflux from vacuoles or endosomes that makes them excellent regulators of intracellular pH (Bassil and Blumwald, 2014; Reguera et al., 2015). In rice, overexpression of *OsNHX1* contributes to Na^+^ compartmentalization in old leaves (Wang et al., 2012) and the expression of *OsNHX1, OsNHX2, OsNHX3*, and *OsNHX5* is regulated differently in rice tissues and is increased by salinity stress (Fukuda et al., 2011).

Enhanced xylem K^+^ loading and translocation is another key determinant of salinity tolerance (Assaha et al., 2017). The high-affinity K^+^ transporters, *OsHAK1*, and *OsHAK5*, are essential for maintaining K^+^-mediated growth and development of rice plants under salinity stress. Overexpression of *OsHAK1* in rice increased K^+^ uptake, maintaining high K^+^/Na^+^ ratio for salt tolerance (Chen et al., 2015a, b). Overexpression of *OsHAK5* increased the K^+^/Na^+^ ratio in the shoots, while knockout of *OsHAK5* decreased shoot K^+^/Na^+^ ratio, resulting in sensitivity to salt stress (Yang et al., 2014). The OsHAK family has many other members that may also have roles in salinity tolerance in rice, which requires further investigation.

Upon exposure to salinity stress, the competitive inhibition of K^+^ uptake by Na^+^ often leads to Na^+^ interfering in many K^+^-dependent processes (Wu et al., 2018). Among the K^+^ channels that have been found in rice that control K^+^ homeostasis during salinity stress is the inward-rectifying K^+^ channel, *OsAKT*s (Fuchs et al., 2004). The maintenance of high K^+^ levels in the cell by preventing of K^+^ efflux is also important for salinity tolerance in plants (Chen et al., 2005). Jayakannan et al. (2013) demonstrated that an influx of Na^+^ in cells depolarizes cell membranes, leading to K^+^ efflux through depolarization-activated K^+^ outward rectifying channels, such as GORK in *Arabidopsis*. In rice, two outward K^+^ rectifying, shaker-like channels have been identified: *OsSKOR* (stelar K^+^ outward rectifier) and *OsGORK*. *OsSKOR* is expressed in root vascular tissues, flowers, and the seed scutellum, while *OsGORK* is expressed to some degree in all tissues (Kim et al., 2015). Liu et al. (2019) showed upregulation of *OsGORK* and *OsAKT1* in tolerant varieties which aids in K^+^ retention in roots when seedlings are exposed to salinity stress. Further elucidation of the roles of this K^+^ channels in salinity stress still needs to be studied.

In plants, there are three primary proton transport proteins: (1) plasma membrane (PM) and (2) vacuolar H^+^-ATPases, which couple ATP hydrolysis with proton transport, and (3) PM and vacuolar H^+^-PPases, which couple pyrophosphate hydrolysis with proton transport (Gaxiola et al., 2007; Fuglsang et al., 2010). Overexpression studies showed that these proton pumps act synergistically together with key ion transporters genes for enhance salinity tolerance. Rice plants overexpressing tonoplast *OsNHX1* and H^+^-pyrophosphatase (*OsVP1*) incurred less damage and had higher photosynthetic activities when exposed to long-term salinity (Liu et al., 2010). It was also shown that the salt-tolerant rice (cv. Pokkalli) shows a rapid and significant increase in expression of vacuolar H^+^ ATPases (*OsVHA*) and *OsHKT2* compared to a sensitive line under salinity stress (Kader et al., 2006).

### New Mechanisms for Salt Tolerance in Wild Rice

Despite selective breeding or better Na^+^ exclusion, even salt-tolerant rice varieties lack tissue tolerance (Yeo and Flowers, 1986; Prusty et al., 2018). This calls for a shift in paradigm in rice breeding for salinity tolerance and the need to target some other (previously underutilized) traits. Such additional mechanisms may include compartmentalization of Na^+^ in roots and older leaves, higher tissue tolerance, efficient ROS production, regulation of stomatal function to control transpiration and passive solute accumulation. Since salinity tolerance is a complex quantitative trait, these mechanisms will not be equally expressed among tolerant cultivated rice. Some salt-tolerant varieties may only contain one or few traits or some may be salt-tolerant at the seedling stage but not at the reproductive stage (Yeo and Flowers, 1986; Ismail and Horie, 2017). Thus, identification of diverse germplasm with high salinity tolerance and integration of all physiological mechanisms should be a long-term goal for salinity stress enhancement in cultivated rice. However, there is a large knowledge gap due to the paucity of research work on breeding salinity tolerance using wild *Oryza* species. Here, we summarize the recent progress on the understanding the salt tolerance mechanisms in wild rice (Supplementary Table S2).

Among the wild rice relatives, *O. rufipogon*, having the same AA genome and the highest compatibility with *O. sativa* (Khush, 1997), is most often used for breeding salinity-tolerant lines. Ganeshan et al. (2016) showed that crosses between *O. sativa* and *O. rufipogon* or *O. nivara* could result in salinity tolerance. Hybrids of *O. rufipogon* × *O. sativa* were found to contain nine quantitative trait loci (QTL) and candidate genes (e.g., HKT1;5, HAK6 and some transcriptional factors) controlling the salt tolerance at the seedling stage (Quan et al., 2018), and 13 of 15 QTLs for salinity tolerance found were from *O. rufipogon* (Tian et al., 2011). Wang et al. (2017) identified 10 QTLs for salt tolerance from introgression lines derived from *O. rufipogon* and *O. sativa* (cv. 93-11). RNA-sequencing found four differentially expressed genes, *OsGH3-2, OsGH3-8, CML15*, and *GEM*, located in these QTL regions from *O. rufipogon*. Recombinant inbred lines (RILs) also derived from a cross of *O. rufipogon* × *O. sativa* showed superior performance with respect to salinity stress (Quan et al., 2018). The cultivated varieties, BRRI Dhan 55(AS996), in Bangladesh and DRR Dhan 40, Jaraya and Chinsurah Nona 2 in India have been released after interspecific hybridization with *O. rufipogon* or *O. nivara* (Supplementary Table S1). Accessions of Indian wild rice germplasm of *O*. *rufipogon* and *O. nivara* were screened for their level of salt tolerance. Linkage disequilibrium (LD) analysis showed significant associations of single nucleotide polymorphisms (SNPs) in key genes for the salt tolerant phenotype (Mishra et al., 2016). In addition, overexpression of the bHLH transcription factors, *OrbHLH001* and *OrbHLH2*, from *O. rufipogon* resulted in salt-tolerant lines of *O. sativa* (Zhou et al., 2009; Li et al., 2010; Chen et al., 2013). *OrbHLH001* positively regulates the inwardly rectifying K^+^ channel, AKT, which implies that tolerance is due to K^+^ homeostasis under high Na^+^ (Chen et al., 2013). However, *O. rufipogon* and *O. nivara* are not halophytes and are unlikely to dramatically improve salt tolerance in cultivated rice in the future, as the lines produced can only survived 8–12 dSm^–1^ (Habiba et al., 2015), which may not be enough if the drastic impact of salinity continues.

Physiological traits for salt tolerance of *O. officinalis* and *O. latifolia* were compared to the salt-sensitive wild species, *O. rufipogon and O. australiensis*. Both *O. officinalis* and *O. latifolia* accumulate a high content of Na^+^ content in leaves and maintain a constant photosynthetic rate under high salinity (Nishizawa et al., 2015, 2016). These studies showed that there are genes related to photosynthesis associated with salinity tolerance in both *O. officinalis* and *O. latifolia* that need to be identified and functionally characterized. The Australian wild rice species, *O. meridionalis* and *O. australiensis*, showed variable tolerance to salinity. Similar to the salt tolerant rice variety, Pokkalli, *O. australiensis*, had less growth inhibition, lower leaf damage, and less biomass reduction (Yichie et al., 2018). Prusty et al. (2018) tested 22 different wild rice species and found that some wild species exhibit superior growth under salinity stress than cultivated rice. *O. coarctata* was found to be the most tolerant wild species, followed by *O. latifolia* and *O. alta* (Prusty et al., 2018). The wild rice species except *O. coarctata* had higher Na^+^ contents in their leaves and low accumulation in roots (Prusty et al., 2018). The potential of the genes conferring salinity tolerance in these wild rice relatives especially in *O. coarctata* remains untapped, and their ability to confer salt tolerance needs further evaluation.

## The Halophytic Wild Rice Species, *Oryza coarctata*

*Oryza coarctata*, formerly known as *Porteresia coarctata*, is an allotetraploid wild rice with high salt and submergence tolerance (Lu and Ge, 2003). *Oryza coarctata* is unique among wild rice species in having the KKLL genome, and phylogenetic analysis suggests that it was one of the basal lineages of rice during grass evolution (Ammiraju et al., 2008). It thrives in coastal areas where it is submerged daily in seawater (Sengupta and Majumder, 2010; Garg et al., 2014). *Oryza coarctata* has shallow roots and waxy leaves, and it has a highly differentiated rhizome system which serves as a salt depository to give tolerance to the daily intrusion of seawater (Sengupta and Majumder, 2010). Propagation of *O. coarctata* is achieved through nodes of the rhizome where the leaf buds are derived (Sengupta and Majumder, 2009). It has been established as an important resource of genes to improve cultivated rice (Garg et al., 2014).

Unicellular trichomes (salt hairs/salt bladders) on *O. coarctata* leaves are the most important morphological adaption to salinized environments (Bal and Dutt, 1986; Flowers et al., 1990). The salt hairs present on leaf adaxial are finger-shaped and can continuously secrete salt-forming crystals on leaf surfaces, a mechanism similar to that known for other halophyte grasses (Sengupta and Majumder, 2009, 2010). Na^+^ concentrations in leaves of *O. coarctata* do not increase during salinity stress (Sengupta and Majumder, 2009), and concentrations of sodium ions washing of the leaves of *O. coarctata* are significantly higher compared to other wild rice relatives, suggesting Na^+^ ion extrusion from the leaf surface (Prusty et al., 2018). Therefore, this is an efficient salinity adaptation strategy to maintain low Na^+^/K^+^ ratio in the cytosol in the leaf mesophyll while still benefiting from using Na^+^ as a cheap osmoticum.

In addition to its morphological adaptations, many physiological adaptions have been reported for *O. coarctata* including the maintenance of a low Na^+^/K^+^ ratio, a high photosynthetic efficiency, maintenance of relative water contents, and efficient functioning of vacuolar H^+^-ATPase under salinity stress (Senthilkumar et al., 2005). *Oryza coarctata* plants maintain a Na^+^:K^+^ ratio at 0.7 in mesophyll cells, 1.3 in roots and 7.3 in root hairs while the environment’s Na^+^:K^+^ ratio is 34.0 (Sengupta and Majumder, 2009). Bal and Dutt (1986) reported similar results and estimated that this species is able to exclude around 22 mmol day^–1^ of Na^+^ per kg of fresh leaves under a 25% seawater salinity treatment. *Oryza coarctata* is similar to most plants in accumulating higher Na^+^ and K^+^ contents in shoots than roots (Bal and Dutt, 1986; Flowers et al., 1990); however, potassium contents barely decrease with increasing salt content (Sengupta and Majumder, 2009). This suggests that *O. coarctata* might have strong, long-distance K^+^ transport and retention mechanisms. Moreover, calcium may be an important factor in salinity tolerance of *O. coarctata* by preventing sodium influx and K^+^ efflux from NSCC channels as well as by other calcium-activated mechanisms (Sengupta and Majumder, 2010).

Recently, a *de novo* assembly of the 665 Mb of the *O. coarctata* genome containing 34,469 predicted protein-encoding genes was first made available by Mondal et al. (2018). Several genes have been sequenced by PCR-based cloning, but only 498 ESTs are available in the NCBI database to date (Chowrasia et al., 2019). The transcription of *OcNHX1* was found to be regulated during the day in seawater and was also rapidly induced by salinity treatment to sequester Na^+^ into vacuoles (Kizhakkedath et al., 2015). Some *O. coarctata* proteins involved in ROS detoxification, photorespiration, cell wall biosynthesis, are highly upregulated under salinity stress in leaves (Sengupta and Majumder, 2009). No systematic study has been conducted to assess salinity stress tolerance in transgenic rice plants expressing genes from *O. coarctata*; thus, much physiological and molecular research remains to determine to decipher high salinity tolerance in this wild rice. Given the presence of stress-responsive genes and distinct morphological adaptation, *O. coarctata* should be considered as a rich genetic resource containing many genes that can be used for rice improvement of resistance to salinity stress.

## Utilizing Wild *Oryza germplasm* for Breeding Salt-Tolerant Rice Varieties

Crop domestication is thought to be one of the most important events that initiated human civilization. Progressive human selection of genetically inherited traits contributed to higher crop production and better-quality during centuries of domestication (Sengupta and Majumder, 2009). However, important traits responsible for tolerance to biotic and abiotic stress are likely to have been lost during the domestication process. Breeders are refocusing on stress tolerance traits found in wild relatives of crops that remain undisturbed in their natural habitats to facilitate the breeding abiotic and biotic stress tolerant crops (Dai et al., 2014; Wang X. et al., 2018). Most research and breeding efforts toward developing salinity tolerant cultivated rice have focused on tolerance mechanisms at the seedling stage (Ismail et al., 2007). However, due to variation in salinity stress response at rice growth stages, tolerance at both the seedling stage and the reproductive stage are currently being taken into consideration by breeders (Ismail et al., 2007; Hossain et al., 2015).

Salinity tolerance is a polygenic trait that involves a complex of responses at cellular, molecular, physiological and whole plant levels and varies depending on the growth stage. Developing salinity-tolerant rice should encompass all these different attributes. Future breeding strategies should prioritize the identification and introgression of candidate genes/QTLs from wild rice species for traits that are not available in cultivated rice. For example, tissue tolerance to salinity is a key trait found in the different wild rice species and consequently molecular markers, RILs, segment substitution lines (CSSL) are being developed for mapping these genes/QTLs for use in breeding and functional genomics. The major goal is to identify several donors contributing to all the traits conferring salinity tolerance and pyramiding them to enhance varietal development.

**FIGURE 4:**
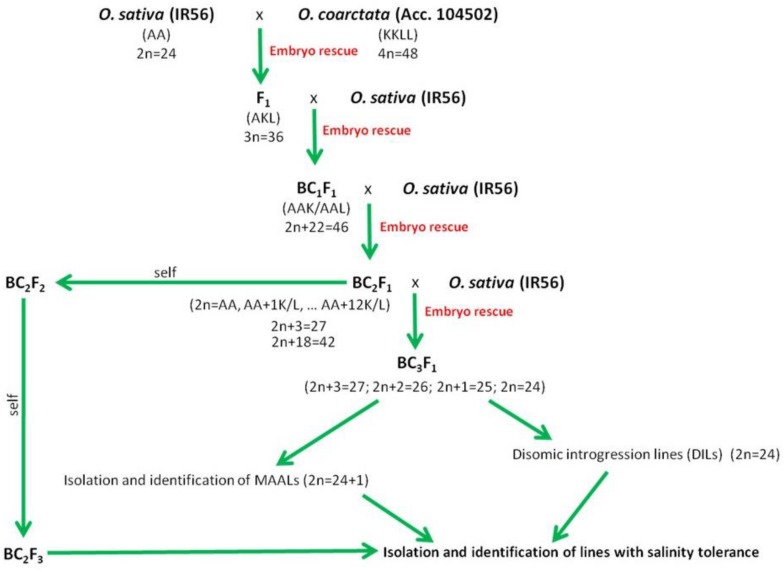
Proposed schematic pipeline on production, isolation, and identification of promising advance backcross of *O. sativa* (IR56) and *O. coarctata* (Acc. 104502) with tolerance to high level of salinity.

In rice, over the course of domestication, introgression of wild rice germplasm from cross compatible species such as *O. rufipogon* and *O. nivara* has occurred, and these species still stand out as active sources of germplasm for enhancement of salinity tolerance (Brar and Singh, 2011). Salinity-tolerant varieties have already been developed and released using these two wild rice species (Chen et al., 2004; Song et al., 2005). The other eight wild rice species with AA genomes can be easily hybridized by conventional breeding (Khush, 1997). However, they are comparatively sensitive to salinity in comparison to halophytic *O. coarctata* that can withstand 40 dSm^–1^ of NaCl. The crossability of *Oryza* species is related to their phylogenetic relationships (Khush, 1997) (Figure 2), and *O. coarctata* is not well utilized for breeding because of its genetic distance to *O. sativa*. Initial attempts to developed hybrid lines have met limited success (Jena, 1994). Crosses made with *O. coarctata* and several salt sensitive cultivars such as IR28, IR36, and Tellahasma suffer drawbacks due to pre- and post-fertilization barriers. Through concerted efforts over the years, advanced introgression lines (BC_*n*_F_*n*_) derived from IR56 × *O. coarctata* that are highly tolerant to salinity stress that can stand 24 dSm^–1^ have been produced at IRRI. A proposed breeding pipeline in producing the salt tolerant varieties using *O. coarctata* is presented in Figure 4. F_1_ progeny are usually sterile due to various incompatibility barriers, but can be used as female parent in backcrossing attempts to produce advanced backcross (BC) progenies and restore fertility through embryo rescue producing an BC_1_F_1_. Subsequently, such plants can be cloned vegetatively for the next round of backcrossing. The same technique can be used to produce the BC_2_F_1_ progenies and so on until advance introgression lines with a high level of salinity tolerance are identified. Individual plants identified as highly tolerant on the rice standard evaluation system (SES) scale (International Rice Research Institute, 2013) can then rescued and subsequently grown for selfing and advancement. Physiological characterization and yield assessment of such progenies to identify the underlying mechanism of tolerance is on-going. The use of bridge species for transferring salinity tolerance to *O. coarctata* should also be taken into consideration. As genetic analysis using RAPD and AFLP markers revealed that *O. coarctata* is closely related to *O. australiensis* (Rangan et al., 2002), it is hypothesized that F_1_ hybrids of *O. coarctata* with *O. australiensis* may facilitate breeding using the former species (Latha et al., 2004).

Recent advances in biotechnology, tissue culture, molecular cytogenetics, comparative genomics, and genomics of rice has enabled gene transfer from wild relatives to cultivate rice. Genetic engineering overcomes barriers to conventional breeding and substantially increases the size of the available gene pool for rice improvement (Key et al., 2008). The most used techniques are electroporation, biolistics, and *Agrobacterium tumefaciens*-mediated transformation and have been used for gene overexpression and silencing in rice (Azhakanandam et al., 2015). The large effort to increase stress tolerance in plants by genetic manipulation has resulted in some significant achievements (Flowers, 2004). In addition, mutation breeding has made a significant contribution toward the production of high yielding and salt stress tolerant rice varieties (Das et al., 2017). Several mutation breeding studies have resulted in rice cultivars with enhanced salinity tolerance, showing many candidate genes related to antioxidants, transcription factors, signaling, ion homeostasis, and transporters to have key roles in salinity tolerance (Hernández, 2019).

The use of wild relatives as a gene pool for new alleles to confer salinity stress tolerance to cultivated species has been successful in other species. For instance, the introgression of the wheat *Nax2* locus, which contains the *TmHKT1;5*-A allele from *Triticum monococcum*, confers enhanced salt tolerance with reduced Na^+^ accumulation in leaves. This has resulted in the wide distribution of germplasm expressing *Nax2* to wheat growers (James et al., 2011; Mickelbart et al., 2015). Wild tomato relatives such as *Lycopersicon cheesmanii* have provided sources of salt tolerance for the cultivated tomato *Lycopersicon esculentum* (Rick and Chetelat, 1995). Considering the different growth environments and habitats for the wild *Oryza* species, it is expected that they also vary in abiotic stress tolerance (Atwell et al., 2014) and will be a rich source of genes.

## Conclusion and Future Perspectives

Wild rice relatives show potential for improving salt tolerance in cultivated rice, and this review proposes incorporating these traits from wild species into cultivated rice is a promising approach toward improving salinity tolerance in rice. It appears to us that the strategy of rice breeding for better Na^+^ exclusion from uptake employed for the last few decades has reached a plateau and cannot deliver any further improvement in salinity tolerance traits in this species. While effectively dealing with Na^+^ cytotoxicity, such selective breeding requires plants to rely on *de novo* synthesis of organic osmolytes for osmotic adjustment. This comes with a high carbon cost (Munns and Gilliham, 2015; Munns et al., 2020) and, hence, massive yield penalties. Thus, we call for a paradigm shift in rice breeding and more efforts toward targeting mechanisms of the tissue tolerance. The possible components may include: (1) efficient internal sequestration of Na^+^ in mesophyll cell vacuoles, with a strong emphasis on control of tonoplast leak channels (Shabala et al., 2019); (2) more efficient control of xylem ion loading to allow rapid osmotic adjustment and turgor maintenance in the shoot (Zarei et al., 2019); (3) efficient cytosolic K^+^ retention in both root and leaf mesophyll cells; and (4) incorporating a possibility of Na^+^ sequestration in external structures such as trichomes (Shabala et al., 2014). This calls for a better utilization of the potential of wild rice where such traits are already present. This also implies developing new screening methods by moving from whole-plant to cell-based phenotyping (Wang H. et al., 2018).

## Author Contributions

Z-HC designed the research. CS and MY conducted the experiments. CS, RV, KJ, and Z-HC prepared the figures and tables. CS, Z-HC, SS, and PH wrote the manuscript with contribution from all authors.

## Conflict of Interest

The authors declare that the research was conducted in the absence of any commercial or financial relationships that could be construed as a potential conflict of interest.
