# Supplementary material for: Back to the Wild: On a Quest for Donors Toward Salinity Tolerant Rice
Source: Front Plant Sci. 2020 Mar 20;11:323. doi: 10.3389/fpls.2020.00323 (PMC7098918; doi:10.3389/fpls.2020.00323)
Supplement: Supplementary file 1 [file Table_1.docx]

**Table S1**. Summary of genome types, chromosome numbers, growth spans, geographical distribution, habitats and number of available accessions of wild Oryza species.

| **Species** | | **Genome group** | **Chromosome Number** | **Growth Span** | **Distribution** | **Usual Habitat** | **Number of Accessions**^a^ |
| --- | --- | --- | --- | --- | --- | --- | --- |
| ***Oryza sativa* complex** | | | | | | | |
|  | *O. sativa* L. | AA | 24 | Annual | Southeast Asia | Upland to deepwater; open | 212,982 |
|  | *O. glaberrima* Stead. | AA | 24 | Annual | West Africa | Upland to deepwater; open | 9,073 |
|  | *O. nivara* S.D.Sharma & Shastry | AA | 24 | Annual | Bangladesh, Cambodia, China, India, Laos, Malaysia, Myanmar, Nepal, Sri Lanka, Thailand, and Vietnam | Seasonally dry | 2064 |
|  | *O. rufipogon* Griff. | AA | 24 | Annual | Australia, Bangladesh, Cambodia, China, India, Indonesia, Laos, Malaysia, Myanmar, Nepal, Papua New Guinea, Philippines, Sri Lanka, Thailand, Vietnam | Shallow swamps; seasonally dry | 1617^b^ |
|  |  | AA | 24 | Perennial | Australia, Bangladesh, Cambodia, China, India, Indonesia, Laos, Malaysia, Myanmar, Nepal, Papua New Guinea, Philippines, Sri Lanka, Thailand, Vietnam | Seasonally deepwater and wet year-round; open | 1617^b^ |
|  | *O. barthii* A. Chev. | AA | 24 | Annual | Benin, Botswana, Burkina Faso, Cameroon, Central Africa Republic,  Chad, Ethiopia, Gambia, Ghana, Guinea, Mali, Mauritania, Namibia, Niger, Nigeria, Senegal, Sierra Leone, Sudan, Tanzania, Zambia | Seasonally dry; open | 405 |
|  | *O. longistaminata* A. Chev. & Roehrich | AA | 24 | Annual | Angola, Benin, Botswana, Burkina Faso, Burundi, Cameroon, Chad, Congo, Democratic Republic of Congo, Ethiopia, Gabon, The Gambia Ghana, Kenya, Madagascar, Malawi, Mali, Martinique, Mozambique, Namibia, Niger, Nigeria, Rwanda, Senegal, Seychelles, Sierra Leone, Somalia, South Africa, Sudan, Tanzania, Uganda, Zambia, Zimbabwe | Seasonally dry to deepwater; open | 309 |
|  | *O. meridionalis* Ng | AA | 24 | Annual | Australia, Indonesia, and Papua New Guinea | Seasonally dry; open | 65 |
|  | *O. glumaepatula* Steud | AA | 24 | Perennial | Bolivia, Brasil, Colombia, Costa Rica, Cuba, Dominica, French Guiana, Guyana, Honduras, Mexico, Panama, Surinam, Venezuela | Inundated areas that is seasonally dry; open | 76 |
| ***O. officinalis* complex** | | | | | | | |
|  | *O. punctata* Kotschy ex Steud. | BB | 24 | Annual | Angola, Benin, Cameroon, Central Africa Republic, Chad, Congo, Ethiopia, Ghana, Kenya, Madagascar, Malawi, Mozambique, Nigeria, Sudan, Swaziland, Tanzania, Uganda, Zambia, Zimbabwe | Seasonally dry; open | 98^c^ |
|  | *O. punctata* Kotschy ex Steud. | BBCC | 48 | Perennial | Angola, Benin, Cameroon, Central Africa Republic, Chad, Congo, Ethiopia, Ghana, Kenya, Madagascar, Malawi, Mozambique, Nigeria, Sudan, Swaziland, Tanzania, Uganda, Zambia, and Zimbabwe | Forest floor; semi shade | 98^c^ |
|  | *O. minuta* JS Presl. ex CB Presl | BBCC | 48 | Perennial | Congo, Ethiopia, Ghana, Kenya, Madagascar, Malawi | Stream sides; semi shade | 86 |
|  | *O. rhizomatis* Vaughan | CC | 24 | Perennial | Mozambique, Nigeria, Sudan, Swaziland, Tanzania, Uganda, Zambia, | Seasonally dry; open | 21 |
|  | *O. eichingeri* Peter | CC | 24 | Perennial | Zimbabwe | Forest floor; shade | 37 |
|  | *O. officinalis Wall ex Watt* | CC | 24 | Perennial | Australia, Bangladesh; Brunei, Cambodia, China, India, Indonesia, Laos, Malaysia, Myanmar, Nepal, Papua New Guinea, Philippines, Thailand, Vietnam | Seasonally dry; open | 317 |
|  | *O. latifolia* Desv. | BBCC | 48 | Perennial | Argentina, Belize, Bolivia, Brazil, Colombia, Costa Rica, Cuba, Dominican Republic, Ecuador, El Salvador, French Guiana, Guatemala, Guyana, Haiti, Honduras, Mexico, Nicaragua, Panama, Paraguay, Peru, Puerto Rico, Surinam, Trinidad and Venezuela | Seasonally dry; open | 86 |
|  | *O. alta* Swallen | CCDD | 48 | Perennial | Belize, Brazil, Colombia, Guyana and Paraguay | Seasonally inundated; open | 27 |
|  | *O. grandiglumis* (Doell.) Prod. | CCDD | 48 | Perennial | Northern Australia | Seasonally dry; open | 52 |
|  | *O. australiensis* Domin | CCDD | 48 | Annual | Northern Australia | Seasonally dry; open | 52 |
|  | *O. brachyantha* A. Chev. & Roehrich | GG | 24 | Annual | Congo, Guinea, Mali, Namibia, Niger, Nigeria, Senegal, Sierra Leone, Sudan, Tanzania, and Zambia | Rock pools; open | 36 |
| ***O. meyeriana* complex** | | | | | | | |
|  | *O. meyeriana* (Zoll. & Moritzi) Baill. | GG | 24 | Perennial | Indonesia, Malaysia, Philippines, and Thailand | Forest floor; shade | 25 |
|  | *O. granulata* Nees & Arn. ex Watt | GG | 24 | Perennial | India, Cambodia, Vietnam, Thailand, southern China, Malaysia, Philippines, Nepal and Sri Lanka | Forest floor; shade | 29 |
| ***O. ridleyi* complex** | | | | | | | |
|  | *O. ridleyi* Hook.f. | HHJJ | 48 | Perennial | Cambodia, Indonesia, Laos, Malaysia, Myanmar, Papua New Guinea, Thailand | Seasonally inundated forest floor; shade | 21 |
|  | *O. longiglumis* Jansen | HHJJ | 48 | Perennial | Indonesia, and Papua New Guinea | Seasonally inundated forest floor; shade | 6 |
| **Unclassified Complex** | | | | | | | |
|  | *O. schlechteri* Pilg. | HHKK | 24 | Perennial | Indonesia and Papua New Guinea | River banks; open | 3 |
|  | *O. coarctata* Roxb. | KKLL | 24 | Perennial | India, Sri Lanka, Bangladesh and Myanmar | Saltmarsh ecosystem; open | 6 |

*Note*: Modified from Khush (1997), Brar and Singh (2011) and Menguer (2017) with recently published papers cited in the text; ^a^Accessions entries per species reported in Genesys Database (<http://www.genesys-pgr.org/>); ^b^Combined accessions entry for annual and perennial *O. ruffipogon*; ^c^Combined accessions entry for annual and perennial *O. punctata*

**Table S2.** Summary of salinity tolerance, key genes, and mechanisms of wild rice relatives and rice varieties developed through interspecific hybridization.

| **Wild rice** | **Tolerance at seedling stage** | **Tolerance at reproductive stage** | **Salinity-responsive genes** | **Mechanisms of adaptation to salinity stress** | **Interspeciﬁc hybridized cultivars** | **References** |
| --- | --- | --- | --- | --- | --- | --- |
| *O. coarctata* | MT | T | *NHX1, PsbR, MT2b, MT, MT2, MT3, L18a, L23a, PP, VPS2.1, IMT1, INO1, NACs, MYBs, WRKYs, OEC, MSP, CP47/PsbB, PsaE, Rubisco activase, chloroplastic precursor of glutamine synthetase, Hsp70, cellulose synthase-like protein* | Na^+^ compartmentilization, Salt exclusion through salt hairs, Unhindered carbon fixation and higher water retention, Higher synthesis of osmoprotectants, Higher ROS detoxification, Higher RUBISCO activation, cell wall synthesis and chaperone functions | - | Sengupta and Majumder, 2009, 2010; Garg *et al.,* 2014; Mishra *et al.,* 2016; Garg *et al.,* 2014 Prusty *et al,* 2018 |
| *O. rufipogon* | HS - MT | MT-T | *OsGH3-2, OsGH3-8, CML15, GEM, LRP1, ABF2, RPK1, DST, HKT2;3, HKT1;5, BADH2, HsfC1B, MIPS1, MIPS2, MYB2, NHX1, NHX2, NHX3, P5CS1, P5CS2, PIP1, SIK1, SOS1, and SOS2* | Na^+^ retrieval from shoot, Higher ROS scavenging activity, Chlorophyll retention | BRRI Dhan 55(AS996), Bangladesh;  Jaraya and DRR Dham 40, India | Ganeshan *et al.,* 2016; Nishizawa et al., 2015, 2016; Tian *et al.,* 2011; Mishra *et al.,* 2016; Wang *et al.,* 2017; Prusty *et al,* 2018 |
| *O. nivara* | HS - MT | T | *HKT2;3, HKT1;5, BADH2, HsfC1B, MIPS1, MIPS2, MYB2, NHX1, NHX2, NHX3, P5CS1, P5CS2, PIP1, SIK1, SOS1, SOS2* | K^+^ transport and Na^+^ retrieval from shoot, Chlorophyll retention | Chinsurah Nona 2, India | Ganeshan *et al.,* 2016; Mishra et al; 2016; Prusty *et al,* 2018 |
| *O. latifolia* | HT | T | *HKT1;4, HKT1;5, SOS1* | Na^+^ retrieval from shoot, Na^+^ exclusion, Na^+^ storage in mature leaves, Chlorophyll retention | - | Nishizawa et al., 2015, 2016*;* Prusty *et al,* 2018 |
| *O. alta* | HT | - | *HKT1;5, SOS1* | Na^+^ retrieval from shoot, Na^+^ exclusion, Chlorophyll retention | - | Prusty *et al,* 2018 |
| *O. grandiglumis* | T | - | *HKT1;5, SOS1* | Na^+^ retrieval from shoot, Na^+^ exclusion | - | Prusty *et al,* 2018 |
| *O. officinalis* | T - HT | - | - | Higher chlorophyll synthesis, photosystem not affected and higher water use efficiency | - | Nishizawa et al., 2016; Prusty *et al,* 2018 |
| *O. rhizomatis* | T | - | - | - | - | Prusty *et al,* 2018 |
| *O. eichingeri* | T | - | - | - | - | Prusty *et al,* 2018 |
| *O. minuta* | T | - | - | - | - | Prusty *et al,* 2018 |
| *O. barthii* | MT | - | - | - | - | Prusty *et al,* 2018 |
| *O. punctata* | MT | - | - | - | - | Prusty *et al,* 2018 |
| *O. australiensis* | S -MT | T | - | Leaf Na^+^ loading, High K^/^Na, Chlorophyll retention | - | Prusty *et al,* 2018; Yichie *et al., 2018* |

*Note*: Tolerance score is based on the varieties being studied in each species by referring to tolerant genotype - Pokkali and sensitive genotype- IR29
